# Necroptosis and *Caspase-2*-Mediated Apoptosis of Astrocytes and Neurons, but Not Microglia, of Rat Hippocampus and Parenchyma Caused by *Angiostrongylus cantonensis* Infection

**DOI:** 10.3389/fmicb.2019.03126

**Published:** 2020-01-23

**Authors:** Hongli Zhou, Zhe Chen, Yanin Limpanont, Yue Hu, Yubin Ma, Ping Huang, Paron Dekumyoy, Minyu Zhou, Yixin Cheng, Zhiyue Lv

**Affiliations:** ^1^Joint Program of Pathobiology, The Fifth Affiliated Hospital, Zhongshan School of Medicine, Sun Yat-sen University, Guangzhou, China; ^2^Key Laboratory of Tropical Disease Control, Sun Yat-sen University, Ministry of Education, Guangzhou, China; ^3^Key Laboratory of Tropical Translational Medicine of Ministry of Education, Hainan Medical University, Haikou, China; ^4^Faculty of Tropical Medicine, Mahidol University, Bangkok, Thailand; ^5^Guangdong Provincial Key Laboratory of Biomedical Imaging, The Fifth Affiliated Hospital, Sun Yat-sen University, Zhuhai, China

**Keywords:** *Angiostrongylus cantonensis*, rat, apoptosis, necroptosis, *caspase-2*

## Abstract

Infection with the roundworm *Angiostrongylus cantonensis* is the main cause of eosinophilic meningitis worldwide. The underlying molecular basis of the various pathological outcomes in permissive and non-permissive hosts infected with *A. cantonensis* remains poorly defined. In the present study, the histology of neurological disorders in the central nervous system (CNS) of infected rats was assessed by using hematoxylin and eosin staining. Quantitative reverse transcription polymerase chain reaction (RT-qPCR), western blot and immunofluorescence (IF) were used in evolutions of the transcription and translation levels of the apoptosis-, necroptosis-, autophagy-, and pyroptosis-related genes. The distribution of apoptotic and necroptotic cells in the rat hippocampus and parenchyma was further detected using flow cytometry, and the features of the ultrastructure of the cells were examined by transmission electron microscopy (TEM). The inflammatory response upon CNS infection with *A. cantonensis* evolved, as characterized by the accumulation of a small number of inflammatory cells under the thickened meninges, which peaked at 21 days post-infection (dpi) and returned to normal by 35 dpi. The transcription levels and translation of *caspase-2, caspase-8, RIP1* and *RIP3* increased significantly at 21 and 28 dpi but decreased sharply at 35 dpi compared to those in the normal control group. However, the changes in the expression of caspase-1, caspase-3, caspase-11, Beclin-1 and LC3B were not obvious, suggesting that apoptosis and necroptosis but not autophagy or pyroptosis occurred in the brains of infected animals at 21 and 28 dpi. The results of RT-qPCR, western blot analysis, IF, flow cytometry and TEM further illustrated that necroptosis and caspase-2-mediated apoptosis occurred in astrocytes and neurons but not in microglia in the parenchyma and hippocampus of infected animals. This study provides the first evidence that neuronal and astrocytic necroptosis and *caspase-2*-mediated apoptosis are induced by *A. cantonensis* infection in the parenchymal and hippocampal regions of rats at 21 and 28 dpi but these processes are negligible at 35 dpi. These findings enhance our understanding of the pathogenesis of *A*. *cantonensis* infection and provide new insights into therapeutic approaches targeting the occurrence of cell death in astrocytes and neurons in infected patients.

## Introduction

The rat lungworm *Angiostrongylus cantonensis*, a food-borne zoonotic parasite, has remained the most common causative agent of neuro-angiostrongyliasis characterized by eosinophilic meningitis or eosinophilic meningoencephalitis worldwide since its first description in 1933 (Chen, 1935; Hu et al., 2018). Various rat species, including *Rattus norvegicus*, *R. rattus*, *R. flavipectus* (Deng et al., 2012), *Bandicota indica*, and *Sigmodon hispidus* (Jarvi et al., 2017; Rael et al., 2018), have been reported as permissive hosts, while mice (*Mus musculus*) (Yamashita et al., 1975), similar to human beings, serve as non-permissive hosts by accidental infection via the ingestion of poorly cooked molluscs, intermediate hosts, or contaminated food or water containing the third-stage larvae (L3) of *A. cantonensis* (Wang et al., 2008; Song et al., 2016). After infection, the L3 larvae migrate to the rat’s central nervous system (CNS), develop into fifth-stage larvae (L5), then travel to the pulmonary arteries and fully mature there. Larvae can migrate from the non-permissive host’s gut to the brain via the circulatory system but cannot develop further (Lu et al., 2018). Rats and mice, which share high nucleotide substitution rates, are rodents that constitute part of the subfamily Murinae of the family Muridae (Blanga-Kanfi et al., 2009; Churakov et al., 2010); they are used in more than 40% of neurological studies (Manger et al., 2008; Krubitzer et al., 2011), are the most widely used animal models for research on vaccine development, drug screening and gene function (Douam and Ploss, 2018), and serve as natural vectors or reservoir hosts for the global transmission and expansion of various infectious pathogens, including bacteria (Du and Wang, 2016), viruses (Forbes et al., 2018; Tanveer et al., 2018), and parasites (Movsesyan et al., 2018). *A. cantonensis* invasion primarily causes negligible or severe eosinophilic meningoencephalitis and meningitis in the CNS of permissive and non-permissive hosts, respectively (Li et al., 2014; Zhang et al., 2017). Although *A. cantonensis* is the most common infectious cause of eosinophilic meningitis in Southeast Asia and the Pacific Basin (Dunn et al., 2019) and distinct pathological outcomes of rats and mice with *A. cantonensis* infection have been observed over the past several decades (Courdurier et al., 1964; Wallace and Rosen, 1969; Uchida et al., 1984), the pathogenicity and pathophysiology of neuro-angiostrongyliasis remain unclear (Morassutti and Graeff-Teixeira, 2012). Determining the molecular events that occur in the rat brain will not only explain the higher tolerance and better adaptation to *A. cantonensis* of permissive hosts than of non-permissive hosts, but also help to better elucidate the pathogenetic mechanisms of *A. cantonensis*.

Infectious diseases caused by bacterial, fungal, and parasitic pathogens induce neurodegenerative disorders and remain a main cause of morbidity and mortality, especially in underdeveloped countries, where parasitic infections are the greatest threat (Shih and Koeller, 2015). Parasitic infections of the host’s CNS are caused by two broad categories of pathogens: single-celled protozoa (*Toxoplasma godii*, *Plasmodium*, *Trypanosoma*, *Leishmania*, *Microsporidium*, and amoebae) and multicellular helminths (*Schistosoma*, *Paragonimus*, *Gnathostoma*, *Echinococcus granulosus*, *Spirometra mansoni*, *Strongyloides stercoralis*, *Toxocara canis*, *A. cantonensis*, and filaria) (Pittella, 2013; Carpio et al., 2016); *A. cantonensis* is the only neurotropic helminth (the larvae migrate to the host’s brain before maturation). The pathogenic mechanisms involved in CNS parasitic infections consist of direct damage caused by the proliferation of protozoan parasites (Schluter and Barragan, 2019) and the physical disruption of tissue by migrating worms (Finsterer and Auer, 2013), while indirect injuries include alterations in the immune status of the CNS (Parlog et al., 2015), the modification of the function and structure of infected cells and the induction of programed cell death of host cells (Zhang et al., 2014; Halonen, 2015; Eugenin et al., 2019). Our previous studies indicated that apoptosis and necroptosis clearly occur in the hippocampal and parenchymal neurons, astrocytes and microglia of infected mice (Luo et al., 2017; Zhang et al., 2017). To further reveal the pathogenesis in permissive hosts infected with *A. cantonensis*, RT-qPCR, western blot analysis and immunofluorescence (IF) were applied to determine apoptosis, necroptosis, autophagy, and pyroptosis levels in the hippocampus and parenchyma of the rats. Flow cytometry and TEM were applied to detect cell death and ultrastructural changes in neurons, astrocytes and microglia of the infected rats.

## Materials and Methods

### Ethics Statement

The Institutional Animal Care and Use Committee of Sun Yat-sen University approved all animal experiments in this study (No. 2017-108). Rats were maintained under specific pathogen-free conditions with unrestricted access to sterilized food and water.

### Parasite Preparation

Infectious L3 of *A. cantonensis* were collected from positive *Biomphalaria glabrata*, which were maintained by the Department of Parasitology, Zhongshan School of Medicine, Sun Yat-sen University. L3 were harvested as described in a previous study (Liu et al., 2017), and the worms were counted under a stereoscopic microscope (SZ650, Cnoptec, Chongqing, China) and prepared for infection.

### Animal Treatment and the Collection of Specimens

Female Sprague-Dawley (SD) rats *(Rattus norvegicus*, 8 weeks old) were supplied by the Experimental Animal Center of Southern Medical University, and all animal procedures were approved by the Institutional Animal Care and Use Committee of Sun Yat-sen University. The rats were housed under specific pathogen-free conditions with a controlled temperature and a 12-h light/dark cycle.

Rats were randomly divided into six groups (15 rats in each group). Each rat (0 dpi) in the experimental groups was orally infected with 100 L3. Fifteen rats were anesthetized with isoflurane and sacrificed at different time points (0, 7, 14, 21, 28, and 35 dpi). The brains of the animals from the control and experimental groups were isolated and washed with 150 μl of minimum essential medium for subsequent use. The hippocampus, parenchyma, epencephalon and brainstem of the animals (3 rats per group) were separated for RT-qPCR and western blot analysis, and the remaining brains were prepared for histopathological examination, IF, flow cytometry, and transmission electron microscopic (TEM) examination.

### Histopathological Examination

The brains of the rats were serially sectioned (5 μm thick), fixed in 4% paraformaldehyde and paraffin-embedded. The tissues were mounted onto slides and stained with hematoxylin–eosin (H&E) for histopathological observation by using an inverted microscope (Leica, Heidelberg, Germany) as previously described (Yan et al., 2018).

### Real-Time Quantitative Polymerase Chain Reaction (RT-qPCR)

Total RNA was extracted from the hippocampus, parenchyma, epencephalon, and brainstem of the rats at each time point using TRIzol Reagent (Thermo Fisher Scientific, Waltham, MA, United States). The concentration of the RNA was determined by a Nano Drop TM spectrophotometer (Thermo Fisher Scientific, Waltham, MA, United States). For RT-qPCR, 4 μg of total RNA was reverse transcribed into complementary DNA (cDNA) by reverse transcriptase. First strand cDNA was synthesized with a Revert Aid First Strand cDNA Kit (Thermo Fisher Scientific, Waltham, MA, United States) according to the manufacturer’s instructions. RT-qPCR was then carried out with SYBR Premix Ex Taq (TaKaRa, Dalian, China) on a Roche LightCycler480 Real-Time PCR platform (Roche Diagnostics, Reinach, Switzerland). The primers used in this experiment are summarized in Table 1. The PCR procedures were performed sequentially as follows: 95°C for 30 s and 35 cycles of 95°C for 5 s and 60°C for 20 s. The melting curve was then measured by heating at 95°C for 1 s, 65°C for 15 s, and 55°C for 30 s. Three independent experiments were performed, and the gene expression levels were analyzed by the 2^–ΔΔ*CT*^ method and normalized to β-actin (Livak and Schmittgen, 2001).

**TABLE 1:** Primers used to amplify the Caspase-1, -2, -3, -6, -8, and -11 genes and the IL-1β, Beclin, LC3B, β-actin, RIP1, and RIP3 genes.

Gene symbol	Forward primer sequence	Reverse primer sequence	References
RIP3	GTGGGATGATGACGACG	TACGACCAGAGGCATACAGG	Liu and Xu, 2016
RIP1	TCCTCGTTGACCGTGAC	GCCTCCCTCTGCTTGTT	
Caspase-2	GGTGATGGTCCTCCCTGTCT	TACTCATCACCAGTGCCAAGC	
Caspase-3	GGACCTGTGGACCTGAAAAA	GCATGCCATATCATCGTCAG	Girling et al., 2018
Caspase-6	ACGTGGTGGATCATCAGACA	GGAGCCGTTCACAGTCTCTC	Girling et al., 2018
Caspase-1	GGAGCTTCAGTCAGGTCCAT	GCGCCACCTTCTTTGTTCAG	Wu et al., 2016
Caspase-11	ATGTGGAGAAGGACTTCATTGC	AGATGACAAGAGCAGGCATGTA	Wu et al., 2016
Caspase-8	CTGGGAAGGATCGACGATTA	TGGTCACCTCATCCAAAACA	
IL-1β	CGACAAAATCCCTGTGGCCT	TGTTTGGGATCCACACTCTCC	Wu et al., 2016
Beclin	TTCAGACTGGGTCGCTTGC	TCCATAGGGAACAAGTCGGTA	
LC3B	CTAACCAAGCCTTCTTCCTCC	AGCCGTCTTCATCTCTCTCGC	
β-actin	GCTACAGCTTCACCACCACA	GCCATCTCTTGCTCGAAGTC	Girling et al., 2018

### Western Blot Analysis

Rat brain tissue samples were lysed with RIPA Lysis Buffer (Thermo Fisher Scientific, Waltham, MA, United States) and quantified by a BCA (bicinchoninic acid) Protein Assay Kit (Beyotime, Wuhan, China). A total of 10 μg of protein from each sample as separated on 12% SDS polyacrylamide gels prior to being transferred to polyvinylidene fluoride membranes (Millipore, Billerica, MA, United States), which were subsequently placed in 5% skimmed milk for 2 h at room temperature. The membranes were then incubated at 4°C overnight with the following primary antibodies: anti-caspase-3, anti-cleaved caspase-3, anti-β-actin, anti-LC3B (Cell Signaling Technology, Danvers, MA, United States), anti-caspase-1, anti-caspase-2 (Abcam, Cambridge, United Kingdom), anti-RIP3 (Santa Cruz, CA, United States), anti-caspase-8 (Cell Signaling Technology, Danvers, MA, United States), and anti-RIP1 (Cell Signaling Technology, Danvers, MA, United States). The membranes were washed three times and then incubated with an HRP (horseradish peroxidase)-labeled secondary antibody (Cell Signaling Technology, Danvers, MA, United States) for 90 min. The signals were detected by using an enhanced chemiluminescence system (Millipore, Burlington, MA, United States) following the manufacturer’s instructions. The density of the bands was analyzed with ImageJ software (National Institutes of Health, Maryland, United States).

### Immunofluorescence (IF)

Consecutive formalin-fixed paraffin-embedded sections of the brains of the animals were subjected to immunostaining as previously described (Zhang et al., 2019). Briefly, PBS (phosphate buffered saline) containing 1% bovine serum albumin (BSA) was used to block the sections for 1 h, followed by incubation with an anti-RIP3 (Santa Cruz, CA, United States) primary antibody overnight at 4°C. After 3 washes with PBS and 0.1% Tween-20 for 5 min, the sections were stained for 1 h with rhodamine-labeled goat anti-mouse lgG (Abways Technology, Shanghai, China), and the cell nuclei were stained with 4’6-diamidino-2-phenylindole for 5 min. Thereafter, the fluorescence intensity of the as were measured under a Leica DM6B fluorescence microscope (Leica, Wetzlar, Germany).

### Flow Cytometry

The hippocampus and parenchyma were separated from the rat brains. After 3 washes, the samples were homogenized on ice into a single-cell suspension with a glass homogenizer and then centrifuged at 1200 rpm for 5 min at 4°C. The cells were resuspended and then incubated with Myelin Removal Beads II (Miltenyi Biotec, Bergisch Gladbach, Germany) to remove the myelin. After that, Fc block was used to block the cells for 30 min on ice before surface staining. Next, the cell surface was stained for 30 min on ice with 50 μl of double distilled water containing 2.5 μl of annexin V PE-cyanine 7, 5 μl of binding buffer, 0.5 μl of Zombie APC-A750 (Biolegend, CA, United States), 1 μl of CD45-BV421 and 1 μl of CD11b-BV711 (BD Biosciences, Franklin Lakes, NJ, United States). The stained cells were washed 3 times, fixed and permeabilized using fixation/permeabilization buffer (BD Biosciences, New Jersey, United States), and then probed with intracellular primary antibodies against GFAP (1:50, BD Biosciences, Franklin Lakes, NJ, United States) and NeuN (1:100, Abcam, Cam-bridge, United Kingdom) in BD Perm/Wash TM buffer. Finally, the cells were transferred into flow tubes and analyzed on a flow cytometer (Beckman Coulter, Atlanta, GA, United States), and 10000 cells per tube were captured for further analysis by CytExpert 2.0 software.

### Transmission Electron Microscopy

For TEM analysis, 4% paraformaldehyde plus 2.5% glutaraldehyde were used to perfuse anesthetized rats. Subsequently, the hippocampus, parenchyma, epencephalon and brainstem were excised immediately, fixed in 2.5% glutaraldehyde overnight and fixed with 1% osmium tetroxide in 100 mM phosphate buffer for 90 min. After dehydration with a graded series of ethanol, the samples were washed and embedded with acetone and Eponate 12 resin. The prepared thin sections (60 nm thick) were fixed on copper and stained with 2% lead citrate solution and uranyl acid. TEM micrographs were taken under a Tecnai G2 Spirit Twin electron microscope (FEI, Hillsboro, OR, United States).

### Statistical Analyses

The statistical analyses were performed using GraphPad Prism 6.0 (GraphPad Software, United States). For significant differences, one-way analysis of variance (ANOVA) followed by the Tukey-Kramer test was applied to analyze differences between the groups. The data in the experiment are displayed as the means ± standard derivation (SD). A *P*-value < 0.05 was considered statistically significant.

## Results

### Histopathological Observation of the Parenchyma and Hippocampus in Rats Infected With *Angiostrongylus cantonensis*

**FIGURE 1:**
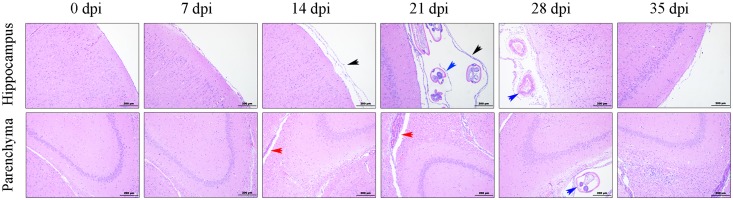
Histopathological alterations in the parenchyma and hippocampus of the rats infected with *A. cantonensis*. The black arrows show inflammatory cells aggregated into the meninges and the thickened meninges. The blue arrows indicate transected in the brains of infected rats. The red arrows point to haemorrhagic lesions in the parenchyma. Magnification: 100×. Scale bars: 200 μm.

To evaluate the histopathological injuries and neuroinflammation in the CNS of the rats infected with *A. cantonensis*, histological examination of the parenchyma and hippocampus from the animals was performed by H&E staining at different time points after infection. In normal rat brains, tissue damage and inflammatory cells were not visible, while the brains of the infected rats exhibited pronounced encephalic lesions (Figure 1), which mainly manifested as severe meningitis, the thickening of the meninges and the accumulation of inflammatory cells (black arrows), at 7, 14, 21, and 28 dpi, with a peak at 21 dpi. Visible haemorrhagic lesions in the parenchyma (red arrows) of the rats were also observed by histopathological examination at 14 and 21 dpi. The histopathology results also showed that the number of infiltrated inflammatory cells and the width of the meninges were obviously decreased in the group at 35 dpi, and no transected worms (blue arrows) were observed in the infected parenchyma (Figure 1).

### Determination of Transcription Levels by RT-qPCR

**FIGURE 2:**
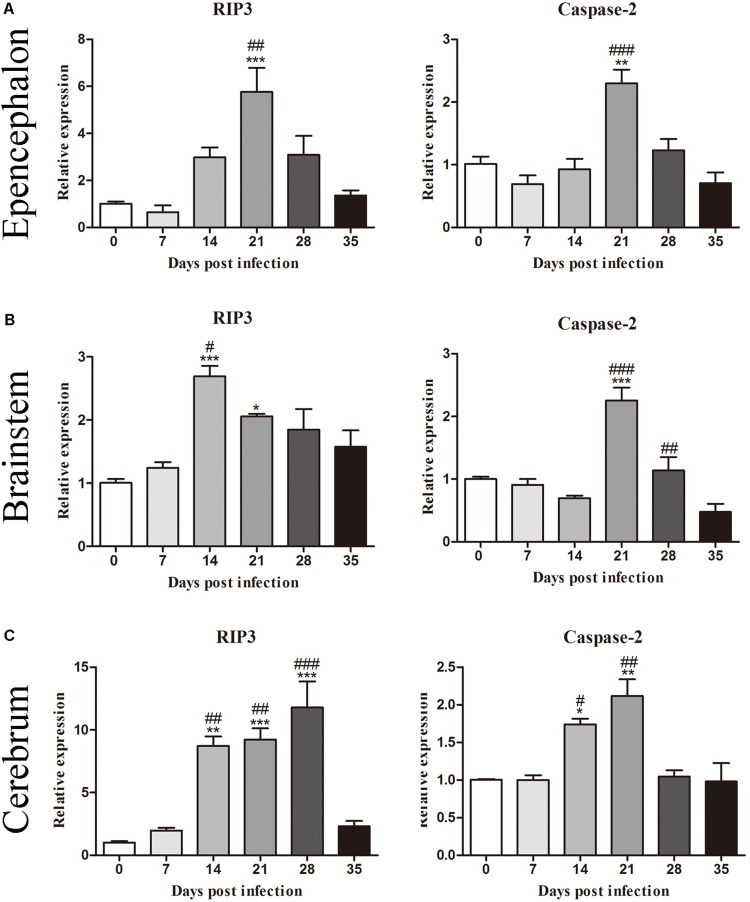
Messenger RNA levels of RIP3 and caspase-2 in the epencephalon **(A)**, brainstem **(B)**, and cerebrum **(C)** of the rats at different time points were determined by RT-PCR (*n* = 3). The bars represent the mean ± SD. ^∗^*p* < 0.05, ^∗∗^*p* < 0.01, and ^∗∗∗^*p* < 0.001 compared with the 0 dpi group; #*p* < 0.05, ##*p* < 0.01, ###*p* < 0.001 compared with the 35 dpi group.

To investigate the mRNA levels of molecules related to cell death, we first determined the levels of *RIP3* and *caspase-2* in the epencephalon, brainstem, and whole brain of the animals from the normal control and experimental groups (Figure 2). The results indicated that the levels of *RIP3* increased significantly after infection, rising nearly fivefold [*F*_(__5_,_12__)_ = 10.67, *P* = 0.001] in the epencephalon (Figure 2A), 2.5-fold [*F*_(__5_,_12__)_ = 10.31, *P* = 0.00185] in the brainstem (Figure 2B) and 10-fold in the whole brain [*F*_(__5_,_12__)_ = 21.5, *P* = 0.0009] by 21 dpi, but decreased sharply at 35 dpi (Figure 2C). Likewise, *caspase-2* mRNA levels increased by approximately 2.1-fold at 21 dpi in the epencephalon [*F*_(__5_,_12__)_ = 12.24, *P* = 0.0022], brainstem [*F*_(__5_,_12__)_ = 20.28, *P* = 0.004], and cerebrum [*F*_(__5_,_12__)_ = 11.34, *P* = 0.0017; Figure 2]. The findings implied that necroptosis and apoptosis occurred in the brains of the infected rats.

**FIGURE 3:**
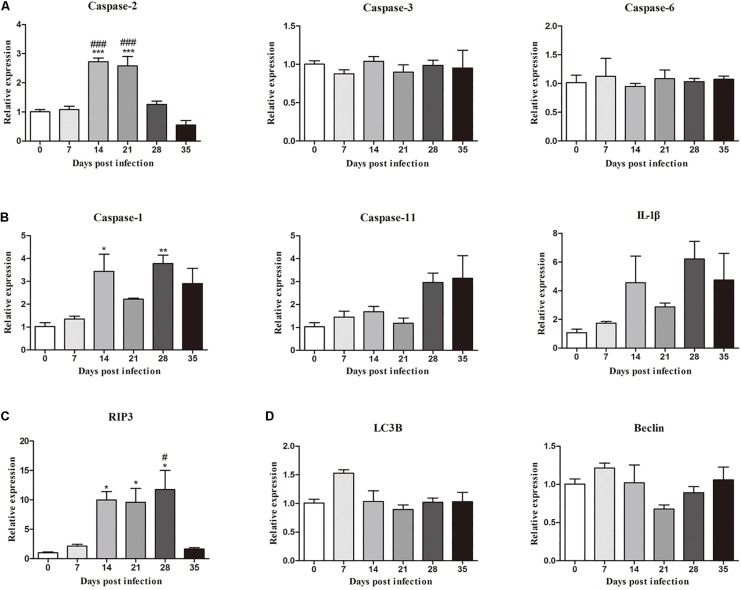
The relative expression of genes related to apoptosis **(A)**, pyroptosis **(B)**, necroptosis **(C)**, and autophagy **(D)** and in the hippocampus of rats infected with *Angiostrongylus cantonensis* at different time points was detected by RT-PCR (*n* = 3). ^∗^*p* < 0.05, ^∗∗^*p* < 0.01, ^∗∗∗^*p* < 0.001 compared with the 0 dpi group; #*p* < 0.05, ###*p* < 0.001 compared with the 35 dpi group.

**FIGURE 4:**
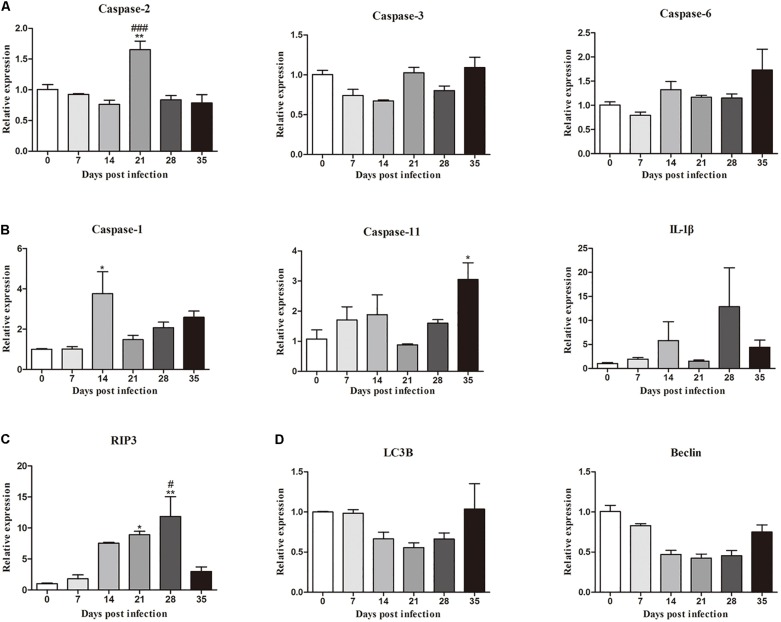
The relative expression of genes related to apoptosis **(A)**, pyroptosis **(B)**, necroptosis **(C)**, and autophagy **(D)** and pyroptosis in the parenchyma of rats infected with *A. cantonensis* at different time points was detected by RT-PCR (*n* = 3). ^∗^*p* < 0.05, ^∗∗^*p* < 0.01 compared with the 0 dpi group; #*p* < 0.05, ###*p* < 0.001 compared with the 35 dpi group.

Moreover, the mRNA levels of genes related to necroptosis (*RIP3*), apoptosis (*caspase-2*, *caspase-3* and *caspase-6*), autophagy (*LC3B* and *Beclin*) and pyroptosis (*caspase-1*, *IL-1*β, and *caspase-11*) in the hippocampus and parenchyma were determined to explore the type of cell death that occurred in the brains of rats infected with *A. cantonensis*. In the hippocampus, the level of *RIP3* increased as the disease progressed, climbing rapidly at 14 dpi [9.85-fold, *F*_(__5_,_12__)_ = 7.777, *P* = 0.0033] and peaking at 28 dpi [11.6-fold, *F*_(__5_,_12__)_ = 7.777, *P* = 0.0043], but decreased strikingly at 35 dpi [*F*_(__5_,_12__)_ = 7.777, *P* = 0.0153] when the worms migrated from the brain to the lungs. The level of *caspase-2* exhibited a similar trend compared to that in the normal control group, being upregulated nearly 3- at 14 [*F*_(__5_,_12__)_ = 26.9, *P* = 0.0002] and 21 dpi [*F*_(__5_,_12__)_ = 26.9, *P* = 0.0004; Figure 3]. Analogously, the expression levels of *RIP3* and *caspase-2* in the parenchyma showed the same dynamic changes as those in the hippocampus (Figure 4). However, as shown in Figures 3, 4, no significant changes in the expression levels of the genes involved in autophagy and pyroptosis (*LC3B*, *Beclin*, *caspase-1*, *IL-1*β, and *caspase-11*) were detected in the hippocampus or parenchyma of the rats from any of the groups. Taken together, the results show that the rats infected with *A. cantonensis* exhibited significantly elevated mRNA levels of *RIP3* and caspase-2 at 14, 21, and 28 dpi but decreased levels at 35 dpi in the hippocampus and parenchyma, whereas there were no obvious differences in the levels of *LC3B*, *Beclin*, *caspase-1*, *IL-1*β or *caspase-11*, suggesting that necroptosis and apoptosis but not autophagy or pyroptosis occurred in the brains of the infected rats.

### Detection of Protein Expression by Western Blot Analysis

**FIGURE 5:**
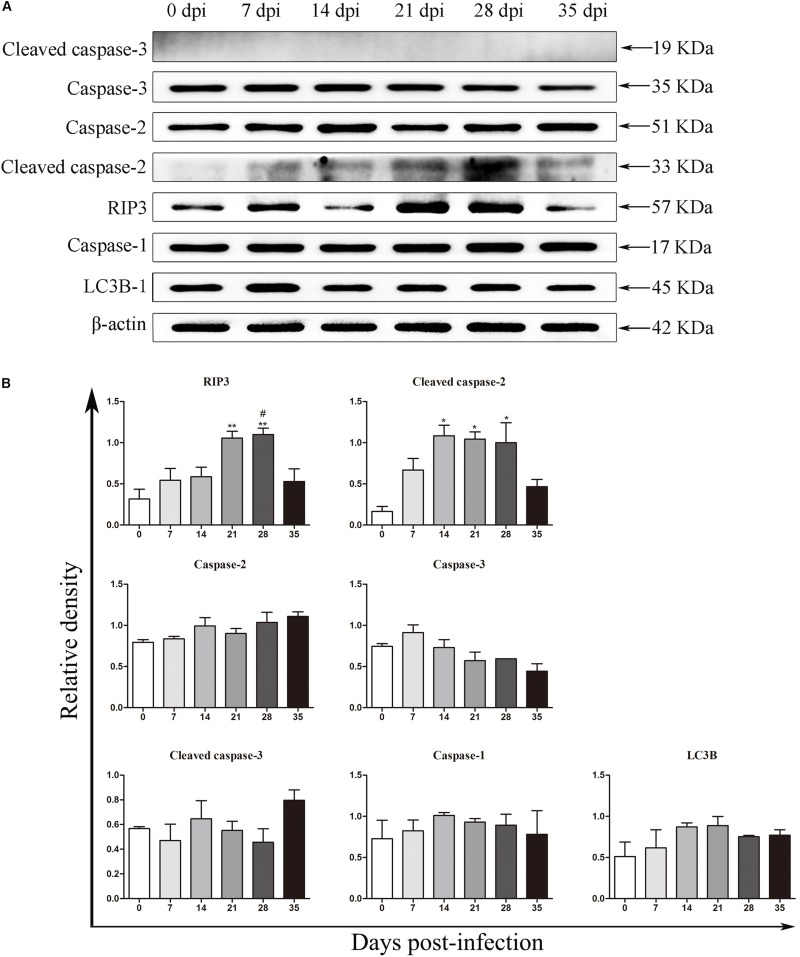
The expression of proteins related to necroptosis, apoptosis, autophagy and pyroptosis in the rat hippocampus was evaluated by western blot analysis **(A)**, and the western blot results were normalized to the level of β-actin in the rat hippocampus **(B)**. ^∗^*p* < 0.05, ^∗∗^*p* < 0.01, and ^∗∗∗^*p* < 0.001 compared with the 0 dpi group; #*p* < 0.05, ##*p* < 0.01 compared with the 35 dpi group.

**FIGURE 6:**
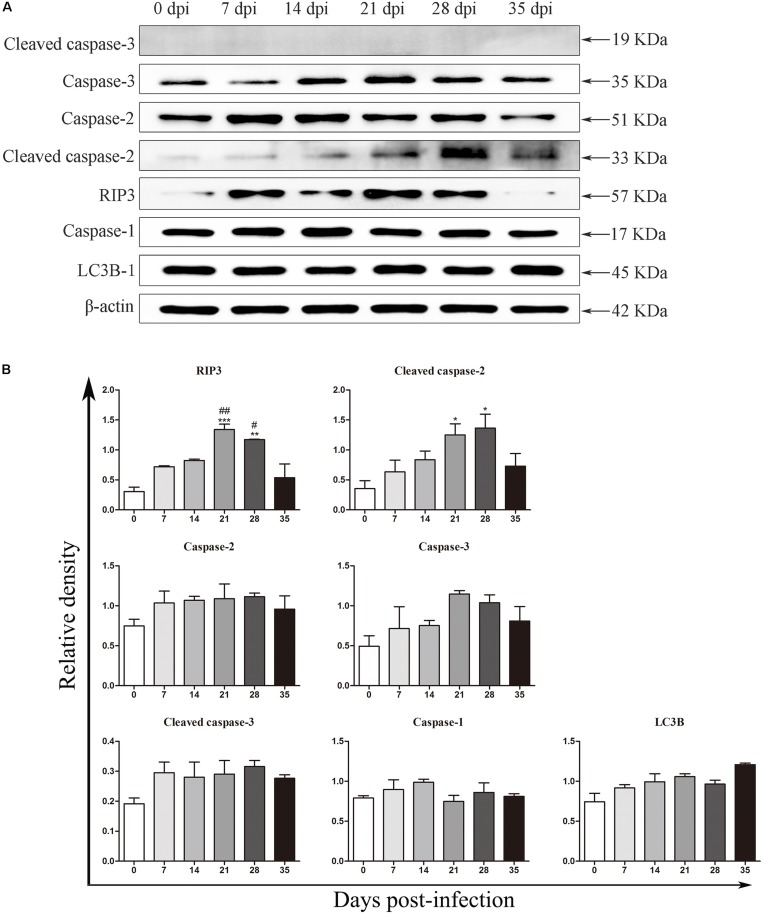
The expression of proteins related to necroptosis, apoptosis, autophagy and pyroptosis in the rat parenchyma was evaluated by western blot **(A)**, and the western blot results were normalized to the level of β-actin in the rat parenchyma **(B)**. ^∗^*p* < 0.05, ^∗∗^*p* < 0.01, ^∗∗∗^*p* < 0.001 compared with the 0 dpi group; #*p* < 0.05, ##*p* < 0.01 compared with the 35 dpi group.

**FIGURE 7:**
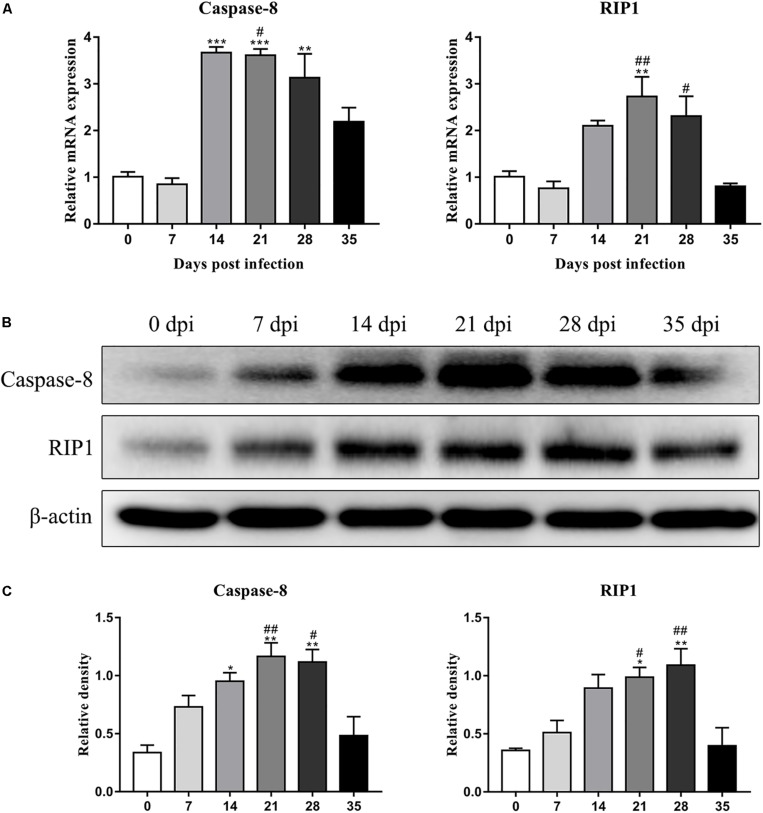
The expression of transcript and protein of caspase-8 and RIP1 in the hippocampus of rats infected with *A. cantonensis* at different time points were analyzed by RT-PCR **(A)** and western blot **(B)**, respectively. The western blot results were normalized to the level of β-actin **(C)**. ^∗^*p* < 0.05, ^∗∗^*p* < 0.01, and ^∗∗∗^*p* < 0.001 compared with the 0 dpi group; #*p* < 0.05, ##*p* < 0.01 compared with the 35 dpi group.

**FIGURE 8:**
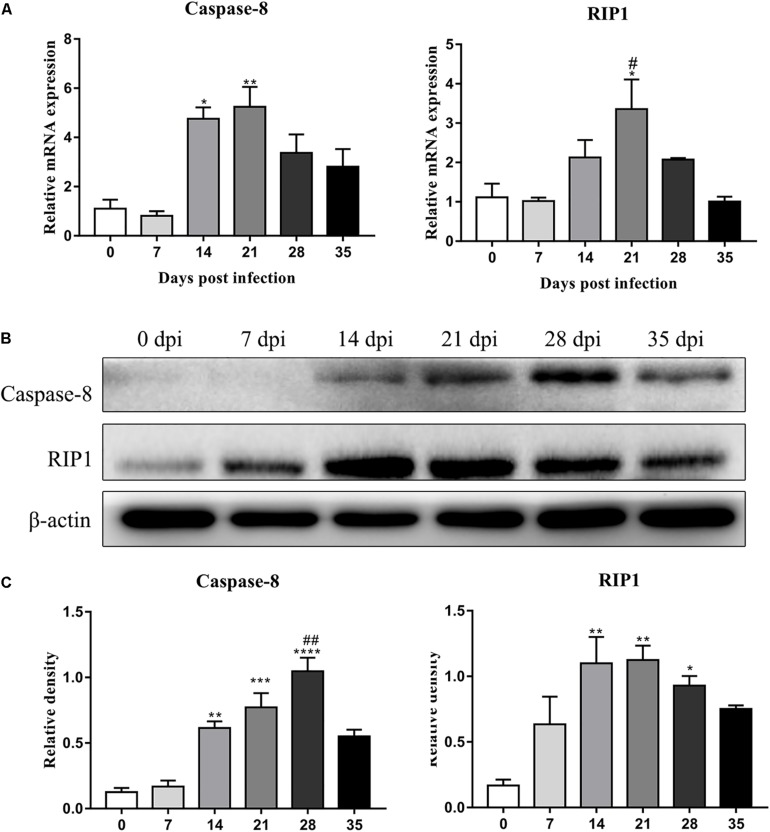
The expression of transcript and protein of caspase-8 and RIP1 in the parenchyma of rats infected with *A. cantonensis* at different time points were analyzed by RT-PCR **(A)** and western blot **(B)**, respectively. The western blot results were normalized to the level of β-actin **(C)**. ^∗^*p* < 0.05, ^∗∗^*p* < 0.01, ^∗∗∗^*p* < 0.001, ^∗∗∗∗^*p* < 0.0001 compared with the 0 dpi group; #*p* < 0.05, ##*p* < 0.01 compared with the 35 dpi group.

The protein levels of key molecules related to necroptosis, apoptosis, autophagy and pyroptosis in the rat hippocampus and parenchyma were further detected by western blot analysis. Consistent with the results of RT-qPCR, the protein level of RIP3 elevated gradually after infection, peaked at 28 dpi [3.3-fold, *F*_(__5_,_12__)_ = 7.102, *P* = 0.0055] and tended to be normal at 35 dpi (Figure 5). Caspase-2 expression showed no significant change, but cleaved caspase-2 protein expression increased dramatically at 14 [6.8-fold, *F*_(__5_,_11__)_ = 5.632, *P* = 0.0171], 21 [6.5-fold, *F*_(__5_,_11__)_ = 5.632, *P* = 0.0229] and 28 dpi [6.3-fold, *F*_(__5_,_11__)_ = 5.632, *P* = 0.0312], with a decrease at 35 dpi in the hippocampus (Figure 5). In the parenchyma, the upregulation of RIP3 and cleaved caspase-2 were also observed at 21 and 28 dpi, with an approximately fourfold change in RIP3 [*F*_(__5_,_12__)_ = 12.34, *P* = 0.0004 and *F*_(__5_,_12__)_ = 12.34, *P* = 0.0017, respectively] and a 3.5-fold change in cleaved caspase-2 [*F*_(__5_,_11__)_ = 4.43, *P* = 0.0426 and *F*_(__5_,_11__)_ = 4.43, *P* = 0.0213, respectively], but both dropped to the normal level at 35 dpi. Meanwhile, the protein levels of caspase-1 and LC3B showed no apparent changes (Figure 6). The apoptosis and necroptosis of rats infected with *A. cantonensis* were further confirmed by the transcripts and translational level of caspase-8 and RIP1. In hippocampus, the results showed that mRNA level of caspase-8 increased rapidly at 14 dpi (3.6-fold, *F*_(5,11)_ = 19.96, *P* = 0.0008) but decreased strikingly at 35 dpi [*F*_(5,11)_ = 19.96, *P* = 0.0889], The level of RIP1 increased as the disease progressed, peaking at 21 dpi [2.7-fold, *F*_(5,11)_ = 9.782, *P* = 0.0093], but decreased strikingly at 35 dpi [*F*_(5,11)_ = 9.782, *P* = 0.0537]. Consistent with the results of RT-qPCR, the protein level of Caspase-8 and RIP1 gradually increased, peaking at 21 dpi [*F*_(5,12)_ = 9.6, *P* = 0.0019] and 28 dpi [*F*_(5,12)_ = 8.042, *P* = 0.0016], respectively, but both tended to be normal at 35 dpi (Figure 7). Similarly, the expression levels of caspase-8 and RIP1 in the parenchyma of rats showed the same dynamic changes as those in the hippocampus (Figure 8). Our findings demonstrated that apoptosis and necroptosis occurred in the rat hippocampus and parenchyma after the invasion of *A. cantonensis* larvae.

### Evaluation of RIP3 Expression and Localization in the Rat Hippocampus and Parenchyma via Immunofluorescence Microscopy

**FIGURE 9:**
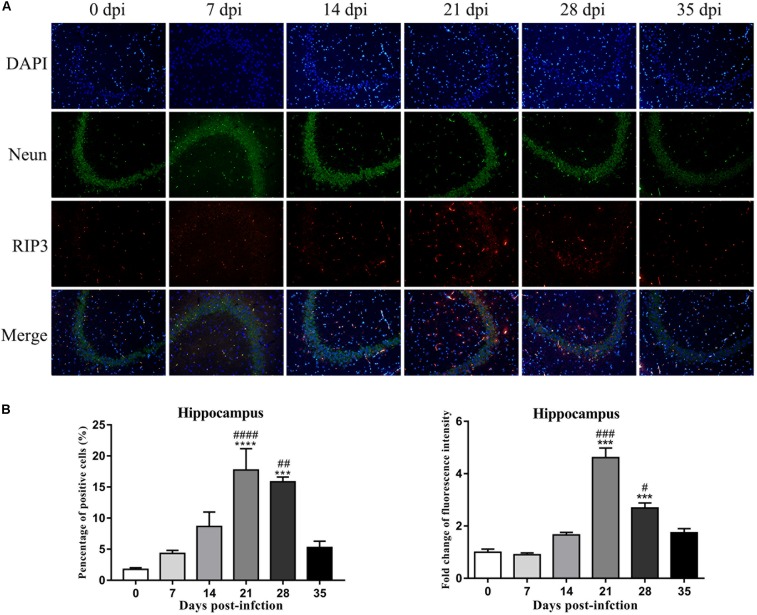
The necroptosis marker RIP3 was analyzed by IF in the rat hippocampus **(A)**, and the percentage of positive cells and the relative fluorescence intensity of RIP3 were evaluated **(B)**. ^∗∗∗^*p* < 0.001, ^∗∗∗∗^*p* < 0.0001 compared with the 0 dpi group; #*p* < 0.05, ##*p* < 0.01, ###*p* < 0.001, *####p* < 0.0001 compared with the 35 dpi group.

**FIGURE 10:**
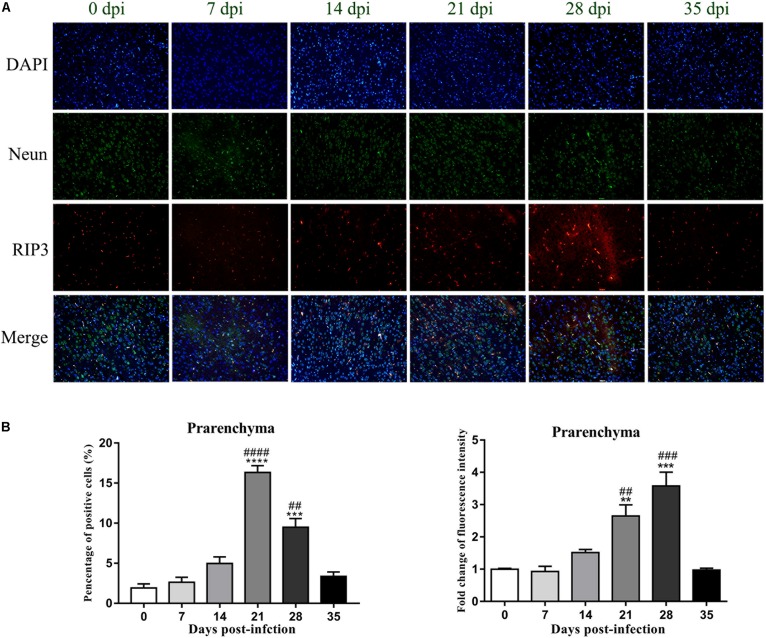
The necroptosis marker RIP3 was analyzed by IF in the rat parenchyma **(A)**, and the percentage of positive cells and the relative fluorescence intensity of RIP3 were evaluated **(B)**. ^∗∗^*p* < 0.01, ^∗∗∗^*p* < 0.001, ^∗∗∗∗^*p* < 0.0001 compared with the 0 dpi group; ##*p* < 0.01, ###*p* < 0.001, ####*p* < 0.0001 compared with the 35 dpi group.

An IF technique was conducted to determine the expression and localization of RIP3 in the infected rat parenchymal and hippocampal cells. As shown in Figures 9, 10, the number of RIP3-positive cells stayed at low levels in the uninfected animals, with percentages of 1.76 ± 0.45% and 1.92 ± 0.89% in the hippocampus and parenchyma, respectively, and no obvious changes in the percentage of positive cells were detected in the rat brain at 7 dpi (4.32 ± 0.87% and 2.62 ± 1.07% in the hippocampus and parenchyma, respectively) and 14 dpi (8.65 ± 4.01% and 4.97 ± 1.42% in the hippocampus and parenchyma, respectively). The number of RIP3-positive cells gradually increased from 7 dpi to 21 dpi, peaking at 17.73 ± 5.95% in hippocampal cells at 21 dpi, but decreased at 35 dpi [(5.27 ± 1.78%) (Figure 9)]. The number of parenchymal RIP3-positive cells reached the highest level (16.33 ± 1.46%) at 21 dpi but reduced to the initial level at 35 dpi (3.35 ± 0.96%) (Figure 10).

To confirm this observation, necroptotic cells were imaged by IF microscopy. There were significant increases of 15.3-fold [*F*_(__5_,_12__)_ = 50.12, *P* < 0.0001] and 7.9-fold [*F*_(__5_,_12__)_ = 21, *P* = 0.0041] in the number of necroptotic cells, which were labeled by RIP3, in infected rat parenchymal and hippocampal cells, respectively, compared with that observed in the normal control group. However, differences between the normal control group, 7, 14, and 35 dpi group were not significant (Figures 9, 10).

Fluorescent double labeling for NeuN (a neuron-specific marker, green) and RIP3 (red) was employed to determine the localization of RIP3 in neurons in the brains of the rats after *A. cantonensis* infection. There were no apparent changes in the number of neurons labeled by NeuN (green) in the hippocampus and parenchyma of the animals with or without infection. The results of dual staining clearly showed that most of the RIP3-positive cells in the hippocampus and parenchyma of the rat brain at 21 and 28 dpi were neurons, and a small number of RIP3-positive cells were other undefined cells, as determined by the merged fluorescence images in Figures 9, 10, which indicated necroptosis occurred in hippocampus and parenchyma of the infected animals.

### Microglia, Astrocytes, and Neurons in the Parenchyma and Hippocampus Showed a Late-Apoptotic/Necrotic Phenotype

**FIGURE 11:**
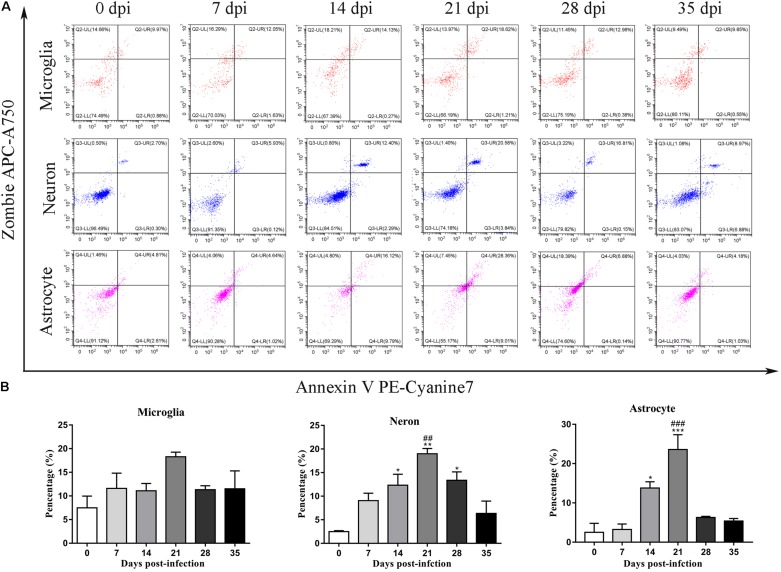
Flow cytometry identified the types of cell death in the hippocampus of rats infected with *A. cantonensis* **(A)**, and the percentage of late-apoptotic and necroptotic microglia, neurons and astrocytes was assessed **(B)**. ^∗^*p* < 0.05, ^∗∗^*p* < 0.01, ^∗∗∗^*p* < 0.001 compared with the 0 dpi group; ##*p* < 0.01, ###*p* < 0.001 compared with the 35 dpi group.

**FIGURE 12:**
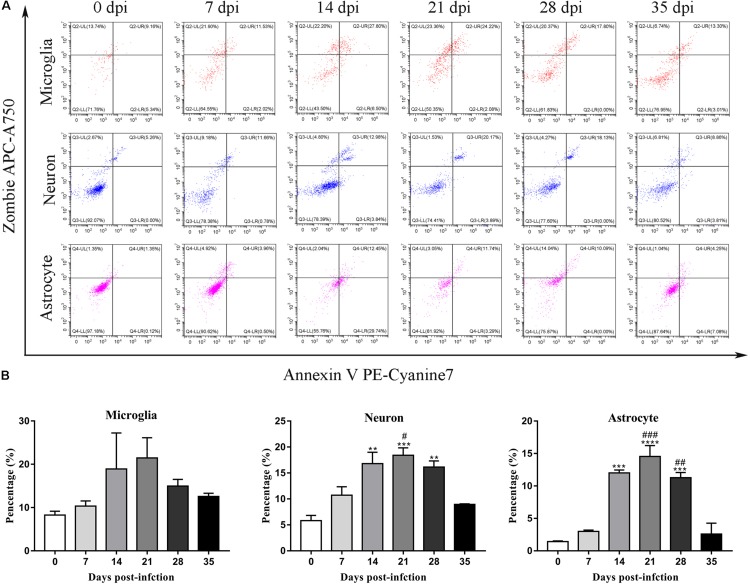
Flow cytometry identified the types of cell death in the parenchyma of rats infected with *A. cantonensis* **(A)**, and the percentage of late-apoptotic and necroptotic microglia, neurons and astrocytes was assessed **(B)**. ^∗∗^*p* < 0.01, ^∗∗∗^*p* < 0.001, ^∗∗∗∗^*p* < 0.0001 compared with the 0 dpi group; #*p* < 0.05, ##*p* < 0.01, ###*p* < 0.001 compared with the 35 dpi group.

To further identify apoptotic and necroptotic cell populations in the brains of the infected rats, we distinguished cells undergoing various types of cell death via corresponding biomarkers by flow cytometry. Similar to our IF microscopy data, the proportion of late-apoptotic/necrotic cells gradually increased at 14, 21, and 28 dpi but decreased at 35 dpi in the hippocampus and parenchyma (Figure 11). The proportion of late-apoptotic/necrotic microglia in the hippocampus and parenchyma peaked at 21 dpi [18.28 ± 1.69%, *F*_(__5_,_11__)_ = 1.96, *P* = 0.1134] and then decreased slightly at 28 dpi (11.33% ± 1.43%). The percentages of neurons and astrocytes rose and peaked at 21 dpi [19.0 ± 1.94%, *F*_(__5_,_10__)_ = 9.347, *P* = 0.0012 and 23.59 ± 6.55%, *F*_(__5_,_10__)_ = 15.25, *P* = 0.0006, respectively]. Similarly, the proportion of late-apoptotic/necrotic neurons and astrocytes in the parenchyma significantly peaked at 21 dpi [18.4 ± 2.52%, *F*_(__5_,_11__)_ = 11.11, *P* = 0.0009 and 14.53 ± 2.922%, *F*_(__5_,_10__)_ = 33.2, *P* = 0.0001, respectively] and then sharply decreased at 35 dpi (8.95 ± 0.13% and 2.60 ± 2.34%, respectively; Figure 12).

### Morphological Changes in Rat Brains Infected With *Angiostrongylus cantonensis*, as Determined by Transmission Electron Microscopy

**FIGURE 13:**
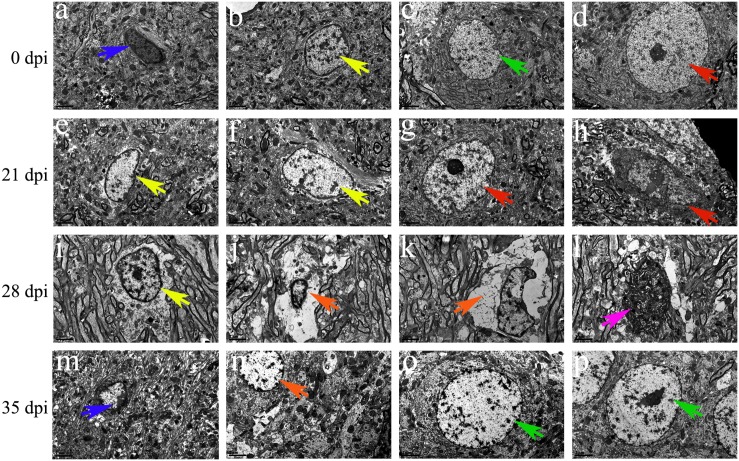
Representative transmission electron micrographs of hippocampal cells from rats from the different groups. The green, blue, yellow and red arrows indicate astrocytes, microglia, oligodendrocytes and neurons, respectively. **(A–D,G,M,O,P)** Normal appearance and ultrastructure. **(E,F,I)** Swollen oligodendrocytes. **(H)** Shrunken neurons. **(J,K,N)** Necrotic cells are indicated by the orange arrows. **(l)** Apoptotic cells are indicated by the purple arrows. Magnification **(A–P)**: 5800×. Scale bars **(A–P)**: 2 μm.

**FIGURE 14:**
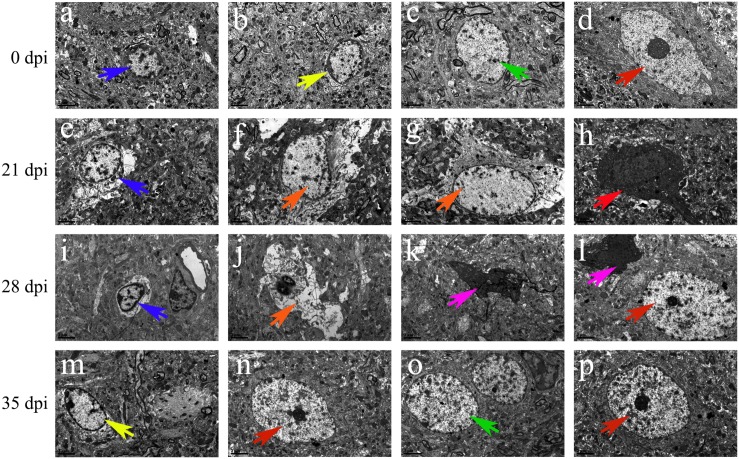
Representative transmission electron micrographs of parenchymal cells from rats from the different groups. The green, blue, yellow, and red arrows indicate astrocytes, microglia, oligodendrocytes and neurons, respectively. **(A–D,N,O,P)** Normal ultrastructure of cells. **(M)** Swollen oligodendrocytes. **(H)** Shrunken neurons. (**E,I)** Swollen microglia. **(F,G,J)** Necrotic cells are indicated by the orange arrows. (**K,L)** Apoptotic cells are indicated by the purple arrows. Magnification **(A–P)**: 5800×. Scale bars **(A–P)**: 2 μm.

Transmission electron microscopy was used to detect morphological changes and the ultrastructure of late-apoptotic/necrotic cells in the hippocampus and parenchyma of rats infected with *A. cantonensis*. Compared to the normal morphology of oligodendrocytes (Figures 13B, 14B), astrocytes (Figures 13C, 14C) and neurons (Figures 13D,G, 14D) at 0 dpi, the morphology of the cells in the 21 and 28 dpi groups was notably different. Oligodendrocytes (Figures 13E,F,I, 14M) and astrocytes with swollen organelles (Figures 13J,K,N, 14F,G,J) and shrunken neurons with broken nuclear membrane (Figures 13H, 14H) were observed in the animals at 21 and 28 dpi and indicated that these cells suffered from necroptosis. Moreover, dense chromatin margination and the patchy nuclear membranes of astrocytes (Figures 13L, 14K) and neurons (Figure 14L) in the infected rats at 21 and 28 dpi revealed that these parenchymal and hippocampal cells underwent apoptosis. No visible morphological differences in microglia (Figures 13A,M, 14A,E,I) were observed between the uninfected and infected animals, and the parenchymal (Figures 14N–P) and hippocampal (Figures 13O,P) cells in the 35 dpi group appeared to have similar morphological characteristics as those in the normal control group. The significantly altered morphology of neurons and astrocytes implied that *A. cantonensis* infection led to apoptosis and necroptosis in the rat brain.

## Discussion

*Angiostrongyliasis cantonensis* infection is a zoonotic disease characterized by neurological manifestations, including eosinophilic meningitis and meningoencephalitis, in non-permissive hosts (OuYang et al., 2012; Zhang et al., 2017). In the definitive host (the rat), however, there appears to be relatively little eosinophilic inflammation and nerve damage in the CNS from 7 dpi to 21 dpi, as detected by H&E staining (Figure 1), compared to the pathological injuries in non-permissive hosts, which was in accord with previous studies (Li et al., 2014; Liu et al., 2017). The histopathology results also indicated that the number of infiltrated inflammatory cells and the width of the meninges were decreased at 28 dpi and tended to be normal at 35 dpi. The reductions in rat brain injury and programed cell death after larval migration from the brain to the lungs implied better adaptation and higher tolerance of rats to *A. cantonensis* infection (Liu et al., 2017).

Cell death, which can be classified as apoptosis, necroptosis, autophagy and pyroptosis in terms of morphological appearance, functional aspects and other features (Melino, 2001; Kroemer et al., 2009), generally progresses as biological ability is lost (Tang and Kang, 2015). The pathogenesis of diseases with enormous social importance, including cancer, HIV, autoimmune diseases and neurological disorders, is closely related to cell death (McConkey et al., 1996; Kaminskyy and Zhivotovsky, 2018). Accumulating studies have demonstrated that Alzheimer’s (Bibi et al., 2014) and Parkinson’s disease (Chen et al., 1995; Inoue, 2006) involve distinct types of cell death. Moreover, neurological dysfunction is thought to occur in CNS upon infection with bacteria (Baldi and Giambartolomei, 2013), viruses (An et al., 1996; Kaul and Lipton, 2006) or parasites (Eugenin et al., 2019) and might occur via apoptosis, necroptosis or autophagy (Tabor-Godwin et al., 2012). HIV-1 infection in AIDS patients has been linked to the loss of CNS neurons through both necrosis and apoptosis (Gelbard et al., 1995; Jones and Power, 2006). The Zika virus elicits the apoptosis of neural progenitor cells and subsequently causes microcephaly in infected infants. *Brucella* lipoproteins, which cause the TNF-α signaling-dependent apoptosis of astrocytes, may be key factors in neurobrucellosis. Cerebral malaria can lead to the apoptosis of neurons and astrocytes through haemozoin (García Samartino et al., 2010), while *Plasmodium falciparum* induces the apoptosis of cerebral microvascular endothelial cells via a perforin-dependent process; both these infections result in neurological disorders (Potter et al., 2006).

In our study, increased expression of RIP3, a specific molecular marker of necroptosis (Vanden Berghe et al., 2014), was first detected in the hippocampal and parenchymal cells of infected rats at 14 and 21 dpi (Figures 3–6), indicating that necroptosis is induced by *A. cantonensis* infection in the rat brain. Additionally, caspase-2, as a member of the caspase family, which is involved in apoptosis (Li et al., 1997), was cleaved and activated in the rat parenchyma at 21 and 28 dpi and in the hippocampus at 14, 21, and 28 dpi (Figures 5, 6), which reveals that obvious apoptosis occurs upon infection with *A. cantonensis*. The results are in accordance with those of our previous study, which showed clear neurological impairments in infected rats, as assessed by using the Morris water maze and neurological function scores (Liu et al., 2017). Surprisingly, RIP3 and cleaved caspase-2 levels were decreased to normal levels at 35 dpi, implying that apoptosis and necroptosis were significantly attenuated after the larvae migrating from the brain to the lungs. Considering the similar dynamic trend of the accumulation of infiltrated inflammatory cells in the brains of rats from 7 dpi to 35 dpi, our findings demonstrate the tight link between apoptosis and necroptosis in the CNS of infected animals.

Caspases play pivotal roles in regulating the apoptotic signaling pathway (Shi, 2002; Fan et al., 2005), and caspase-2 highly evolutionarily conserved and is widely distributed in various cell and tissue types (Bouchier-Hayes, 2010). Some caspases can initiate apoptosis, while caspase-2 tends to be an apoptosis inducer (Bonzon et al., 2006) that is upstream of the mitochondrial pathway and is related to the activation of pro-caspase-9 (Guo et al., 2002; Read et al., 2002). Caspase-2 has also been implicated in pathologies associated with neurodegenerative disorders such as Huntington’s disease, Alzheimer’s disease and aging (Shimohama et al., 1999; Troy et al., 2000; Hermel et al., 2004). Caspase-3 and -6, the effector caspases in apoptosis, are highly homologous and mediate morphological and biochemical alterations (Riedl and Shi, 2004; Mazumder et al., 2008). Furthermore, pro-caspase-3 can be positively activated by caspase-6 (Boatright and Salvesen, 2003; Day et al., 2009). Upon activation, caspase-3 and caspase-6 ultimately lead to extensive injury of the nervous system by mediating cell death under chronic neurodegenerative conditions (Eldadah and Faden, 2000). The various caspases that are involved in hippocampal and parenchymal apoptosis in permissive hosts (caspase-2, Figures 3–6) and non-permissive hosts (caspase-3 and caspase-6) (Li et al., 2014) upon *A. cantonensis* infection might help to uncover the underlying mechanism of the distinct pathological outcomes in infected rats and mice. Additionally, novel drugs, such as acetylcysteine, that target apoptosis and necroptosis signaling pathways in combination with anthelmintics may serve as alternative therapies for the treatment of *A. cantonensis* infection since some of them have been approved by the United States Food and Drug Administration for the treatment of cancer, neuroblastoma and autoimmune and inflammatory diseases (Ocker and Höpfner, 2012; Denisenko et al., 2018; Kaminskyy and Zhivotovsky, 2018; Valter et al., 2018).

As the sole immune cells in the brain, microglia play essential roles in maintaining brain functions by plays sentinel, housekeeping function and defense roles (Wolf et al., 2017; Hickman et al., 2018). Microglia strongly influence the pathologic outcomes in non-infectious pathological states (Ramirez et al., 2017; Ikegami et al., 2019) and acts as pivotal mediators of neuroinflammation via the release of cytokines, chemokines, and growth factors in various infectious diseases to regulate innate immunity and participate in adaptive immune responses in CNS tissue (Colonna and Butovsky, 2017). Our previous work indicated that microglial activation, which is considered a main target for therapeutic interventions for a variety of CNS diseases (Heppner et al., 2005; Garden and Möller, 2006), appears to be one of the checkpoints in CNS inflammation caused by *A. cantonensis* infection (Zhao et al., 2013; Wei et al., 2015), and the administration of minocycline, an inhibitor of microglial activation, is effective in treating mice infected with *A. cantonensis* (unpublished data). Interestingly, as described in our previous study, apoptosis, and necroptosis of microglia are observed in infected mice (Zhang et al., 2017), but not in rats after infection (Figure 12), which suggests that microglia exhibit resistance to apoptosis and necroptosis or a lack of effective inducers of microglial cell death in infected rats.

## Conclusion

In summary, the present work is the first to illustrate that *A. cantonensis* infection, particularly at 21 dpi, can significantly lead to caspase-2-mediated apoptosis and necroptosis of neurons and astrocytes in the hippocampus and parenchyma of the rat brain, which is strikingly attenuated at 35 dpi. Our findings might be helpful for better understanding *A. cantonensis* pathogenesis and may provide potential therapeutic targets for the treatment of angiostrongyliasis.

## Data Availability Statement

All datasets generated for this study are included in the article/supplementary material.

## Ethics Statement

The animal study was reviewed and approved by the Institutional Animal Care and Use Committee of Sun Yat-sen University.

## Author Contributions

ZL conceived and designed the study. ZL and HZ drafted the manuscript. HZ and ZC carried out the experiments. YL, PH, YH, YM, and YC participated in data analysis. PD and MZ participated in study design, technological guidance, and coordination. All authors read and approved the final manuscript.

## Conflict of Interest

The authors declare that the research was conducted in the absence of any commercial or financial relationships that could be construed as a potential conflict of interest.
